# Men's and Women's World Championship Marathon Performances and Changes With Fatigue Are Not Explained by Kinematic Differences Between Footstrike Patterns

**DOI:** 10.3389/fspor.2020.00102

**Published:** 2020-08-06

**Authors:** Brian Hanley, Athanassios Bissas, Stéphane Merlino

**Affiliations:** ^1^Carnegie School of Sport, Leeds Beckett University, Leeds, United Kingdom; ^2^School of Sport and Exercise, University of Gloucestershire, Gloucester, United Kingdom; ^3^Development Department, World Athletics, Monte Carlo, Monaco

**Keywords:** athletics, endurance, performance, running, videography

## Abstract

World-class marathon runners make initial contact with the rearfoot, midfoot or forefoot. This novel study analyzed kinematic similarities and differences between rearfoot and non-rearfoot strikers within the men's and women's 2017 IAAF World Championship marathons across the last two laps. Twenty-eight men and 28 women, equally divided by footstrike pattern, were recorded at 29.5 and 40 km (laps 3 and 4, respectively) using two high-definition cameras (50 Hz). The videos were digitized to derive spatiotemporal and joint kinematic data, with additional footage (120 Hz) used to identify footstrike patterns. There was no difference in running speed, step length or cadence between rearfoot and non-rearfoot strikers during either lap in both races, and these three key variables decreased in athletes of either footstrike pattern to a similar extent between laps. Men slowed more than women between laps, and overall had greater reductions in step length and cadence. Rearfoot strikers landed with their foot farther in front of the center of mass (by 0.02–0.04 m), with non-rearfoot strikers relying more on flight distance for overall step length. Male rearfoot strikers had more extended knees, dorsiflexed ankles and hyperextended shoulders at initial contact than non-rearfoot strikers, whereas female rearfoot strikers had more flexed hips and extended knees at initial contact. Very few differences were found at midstance and toe-off. Rearfoot and non-rearfoot striking techniques were therefore mostly indistinguishable except at initial contact, and any differences that did occur were very small. The spatiotemporal variables that differed between footstrike patterns were not associated with faster running speeds and, ultimately, neither footstrike pattern prevented reductions in running speeds. The only joint angle measured at a specific gait event to change with fatigue was midswing knee flexion angle in men. Coaches should thus note that encouraging marathon runners to convert from rearfoot to non-rearfoot striking is unlikely to provide any performance benefits, and that training the fatigue resistance of key lower limb muscle-tendon units to avoid decreases in step length and cadence are more useful in preventing reductions in speed during the later stages of the race.

## Introduction

The marathon (42.195 km) is the longest running race at major events such as the Olympic Games and World Athletics Championships. The marathon is a particularly difficult event to succeed in because glycogen depletion normally occurs after approximately 30 km (Jeukendrup, 2011) with a consequent increase in energy dependence on lipids (O'Brien et al., 1993). This reliance on a slower source of energy during the late stages of the marathon usually results in considerable deceleration over the last 10–15 km that affects even world-class runners (Hettinga et al., 2019), and is known colloquially as “hitting the wall” (Buman et al., 2009). Previous studies on the effects of fatigue on predominantly non-elite marathon runners showed that decreases in step length, rather than cadence, were responsible for this decrease in speed (Buckalew et al., 1985; Chan-Roper et al., 2012). It is possible that world-class marathon runners have, by contrast, developed strategies in training to cope with or prevent the onset of fatigue. Alongside physiological (Stellingwerff, 2012) and pacing strategies (Deaner et al., 2019), athletes could potentially improve marathon performances by incorporating particular biomechanical principles with regard to running form and technique (Pizzuto et al., 2019), and therefore try to prevent such dramatic changes in speed during competition.

Adopting a particular footstrike pattern is one aspect of technique that could possibly lead to better long-distance running performances. Marathon runners are predominantly rearfoot strikers (RFS) at both world-class (Hanley et al., 2019) and recreational standards (Larson et al., 2011), although the proportion of midfoot strikers and forefoot strikers in a world-class sample was higher than amongst recreational runners (Hanley et al., 2019). Non-rearfoot striking (NRFS), which encompasses both midfoot and forefoot striking, arises from an anterior footstrike position that theoretically stores and releases greater elastic energy in the Achilles tendon and foot arches than RFS (Perl et al., 2012), and is practiced by most athletes competing in the shorter middle-distance events over 800 and 1500 m (Hayes and Caplan, 2012). Contact times were shorter in the faster NRFS athletes (Hayes and Caplan, 2012), and this might be related to how less time spent in contact was similarly associated with faster half-marathon running (Gómez-Molina et al., 2017; Ogueta-Alday et al., 2017). However, its lower incidence amongst elite-standard marathon runners might occur because running economy during RFS is similar to NRFS (Ardigò et al., 1995; Gruber et al., 2013), and because carbohydrate oxidation rates were indeed found to be higher during forefoot striking than RFS (Gruber et al., 2013). Additionally, many marathon runners who are NRFS during the first half of the race change to RFS in the second half (Larson et al., 2011; Hanley et al., 2019), possibly because continuous NRFS requires increased ankle plantarflexor work and can lead to considerable fatigue in the contractile properties of those key leg muscles (Peltonen et al., 2012; Baggaley et al., 2017).

One potential biomechanical advantage of landing with an NRFS pattern is that the foot lands closer to the whole body center of mass (CM), with a theoretical reduction in braking forces during early stance (Lieberman et al., 2015; Moore, 2016). At an equal running speed, this shorter distance from landing foot to CM should result in reduced step lengths and higher cadences in NRFS (Goss and Gross, 2013), and is achieved through greater knee flexion and ankle plantarflexion at initial contact (Almeida et al., 2015). These greater lower limb angles in turn lead to less overstriding in NRFS (i.e., the ankle lands more directly under the knee), with potential benefits including more limb compliance at the ankle and knee (Lieberman, 2014). Such differences in technique have been inferred by coaches to mean that NRFS could provide benefits such as improved performance and reduced injury risk (Abshire and Metzler, 2010; Anderson, 2018), but Williams (2007) reported that a female marathon runner with a forefoot strike experienced injury because the increased knee flexion that compensated for subtalar pronation during stance increased the stress on the Achilles tendon and foot arches. Notably, many previous experimental studies on kinematic differences between RFS and NRFS were conducted for short durations only using treadmills (Goss and Gross, 2013), analyzed men only (Shih et al., 2013), included a barefoot condition that is not normal in competition (Perl et al., 2012) or instructed habitual RFS runners to adopt a non-habitual NRFS pattern (Ardigò et al., 1995). Furthermore, no studies have compared men and women, or athletes of different footstrike patterns, with regard to how their gait kinematics change during the final stages of a world-class marathon, when the race outcome is often decided. Therefore, a novel study that analyzes well-trained men and women running in a fatiguing competition with their natural footstrike patterns and own footwear will provide athletes and coaches with robust evidence of similarities and differences between RFS and NRFS that could inform training practices, such as running drills. Such information could also be used by coaches to decide whether to encourage their athletes to change footstrike pattern, especially with regard to the effects of fatigue during the latter stages of the marathon.

No previous research has examined the spatiotemporal or joint kinematic differences between RFS and NRFS in world-class athletes and, furthermore, neither sex-based differences nor the potential effects of fatigue have been analyzed in competition. The aim of this novel study was to analyze spatiotemporal and joint kinematic variables in male and female marathon RFS and NRFS runners across the last two 10.5-km laps at the 2017 IAAF World Championships. Based on previous research, it was hypothesized that RFS would have longer steps and lower cadences than NRFS, resulting from differences in knee and ankle angles at initial contact. It was also hypothesized that those differences found between RFS and NRFS would be similar for men and women, but that men would have greater absolute magnitudes for spatial values (e.g., flight distance), although not when normalized as a proportion of step length. It was further hypothesized that running speed and associated spatiotemporal variables would decrease between the second-last and last laps because of fatigue, but that any differences between RFS and NRFS would be consistent across the last two laps.

## Materials and Methods

### Research Approval

Data were collected as part of the London 2017 World Championships Biomechanics Research Project. The use of those data for this study was approved by the IAAF (since renamed World Athletics), who own and control the data, and locally through the Leeds Beckett University research ethics procedures.

### Participants

Twenty-eight men (39% of the 71 finishers) and 28 women (36% of 78 finishers) were analyzed in their respective races, held on the same day and on the same course. Personal record (PR) and finishing times were obtained from the open-access World Athletics website (World Athletics, 2019, 2020) for competitors in both races. Fifty percent of the 28 athletes analyzed in each race were RFS and 50% were NRFS.

### Data Collection

The men's and women's marathon races were held on a course that consisted of four approximately 10.5 km loops, with the remaining distance comprising a section that led from the start/finish line to the beginning of the loop. A section of straight, wide road near the end of the loop was chosen for video capture so that data collection occurred at approximately 29.5 and 40 km. Two stationary Sony NXCAM HXR-NX3 full high-definition digital cameras (Sony, Tokyo, Japan) were placed on one side of the course, approximately 45° and 135° to the plane of motion, respectively. Each camera was approximately 8 m from the path of the runners. The sampling rate for each camera was 50 Hz, the shutter speed was 1/1250 s, and the resolution was 1920 × 1080 px. The reference volume was 7.50 m long, 3.08 m wide and 1.99 m high. The reference poles were placed so that the 3.08 m width coincided with the path taken by all analyzed runners (marked as the shortest possible route with blue paint by the event organizers). The poles were aligned vertically with the use of a spirit level and plumb line with calibration procedures conducted before and after competition. This approach produced a number of non-coplanar control points and facilitated the construction of specific global coordinate systems.

The procedures used to collect data for the analysis of footstrike patterns have been described previously (Hanley et al., 2019). In brief, two Casio Exilim high-speed cameras (Casio, Tokyo, Japan) were positioned approximately 0.30 m above the running surface on tripods with their optical axes perpendicular to the running direction. The sampling rate for each camera was 120 Hz, the shutter speed was 1/1000 s, and the resolution was 640 × 480 px.

### Data Analysis

The video files were imported into SIMI Motion (SIMI Motion version 9.2.2, Simi Reality Motion Systems GmbH, Germany) and manually digitized by a single experienced operator to obtain spatiotemporal and kinematic data. An event synchronization technique (synchronization of four critical instants: right initial contact, right toe-off, left initial contact and left toe-off) was applied to synchronize the two-dimensional coordinates from each camera. Digitizing started 10 frames before the beginning of the first identified gait event (i.e., initial contact or toe-off) and completed 10 frames after the same event during the next gait cycle to provide padding during filtering (Smith, 1989); the start of the next gait cycle was digitized to identify the succeeding step and to provide padding. Therefore, for each athlete, one gait cycle was digitized for each of the last two laps. Each file was first digitized frame-by-frame and, upon completion, adjustments were made as necessary using the points-over-frame method (Bahamonde and Stevens, 2006), where each point was tracked through the entire sequence. The magnification tool in SIMI Motion was set at 400% to aid identification of body landmarks. The 3D Direct Linear Transformation algorithm (Abdel-Aziz et al., 2015) was used to reconstruct the three-dimensional coordinates from each camera's x- and y-image coordinates. De Leva's 14-segment body segment parameter model (de Leva, 1996) was used to obtain data for the CM and for several body segments of interest. Occasionally, dropout occurred where joint positions were not visible, and estimations were made by the operator. Two separate approaches were taken for removing noise (Giakas and Baltzopoulos, 1997): a cross-validated quintic spline smoothed the raw data before coordinate calculations, whereas a recursive second-order, low-pass Butterworth digital filter (zero phase-lag) filtered the same raw data and first derivatives were subsequently obtained. The cut-off frequencies were calculated using residual analysis (Winter, 2005) and ranged between 4.0 and 7.5 Hz.

To ensure reliability of the digitizing process on the speed and spatiotemporal data, repeated digitizing (two trials) of one running sequence (a single digitized gait cycle from one lap of one runner) was performed with an intervening period of 48 h. Three statistical methods for assessing reliability were used: 95% limits of agreement (LOA), coefficient of variation (CV) and intraclass correlation coefficient (ICC) (Atkinson and Nevill, 1998). The data for each tested variable were assessed for heteroscedasticity by plotting the standard deviations (SD) against the individual means of the two trials (Atkinson and Nevill, 1998). If the data exhibited heteroscedasticity, a logarithmic transformation of the data (log_e_) was performed before the calculation of absolute reliability measures (Bland and Altman, 1986). The LOA (bias ± random error), CV and ICC (3,1) values for CM horizontal speed were 0.000 ± 0.015 m/s, ± 0.13%, and 1.00, respectively; for the right foot horizontal coordinates 0.001 ± 0.003 m, ± 0.04%, and 1.00, respectively; and for the left foot horizontal coordinates 0.001 ± 0.006 m, ± 0.08%, and 1.00, respectively. The results that relate to the most important spatiotemporal variables therefore showed minimal systematic and random errors, and confirmed the high reliability of the digitizing process with regard to the overall group of athletes. In addition, because the hip joint center markers were used to calculate seven angles between them, the effect of misplacing body landmarks was measured by altering both hip joint center markers laterally by one pixel for one man and one woman. The difference in angle magnitudes between the original and altered files was measured and the root mean square difference (RMSD) calculated for one complete gait cycle; the mean RMSD was 0.2° (± 0.1) for both participants.

Footstrike patterns were defined using the foot position at initial contact with the ground using the methods of Hasegawa et al. (2007) as either: RFS (the heel contacted the ground first without simultaneous contact by the midfoot or forefoot), midfoot striking (the heel and midfoot, or occasionally the entire sole, contacted the ground together) or forefoot striking (the forefoot/front half of the sole contacted the ground first with a clear absence of heel contact). As there were very few forefoot strikers in either race (Hanley et al., 2019), midfoot and forefoot strikers have been combined as NRFS. Half of the athletes analyzed on lap 3 in each race were RFS, and the other half were NRFS (i.e., *N* = 14 of each footstrike pattern). All men analyzed were consistently either RFS or NRFS on both laps, with one of the NRFS men adopting forefoot striking on lap 3 and midfoot striking on lap 4; however, two women switched from NRFS on lap 3 to RFS on lap 4. Of the other women, one of the NRFS was a forefoot striker on both laps.

**Figure 1:**
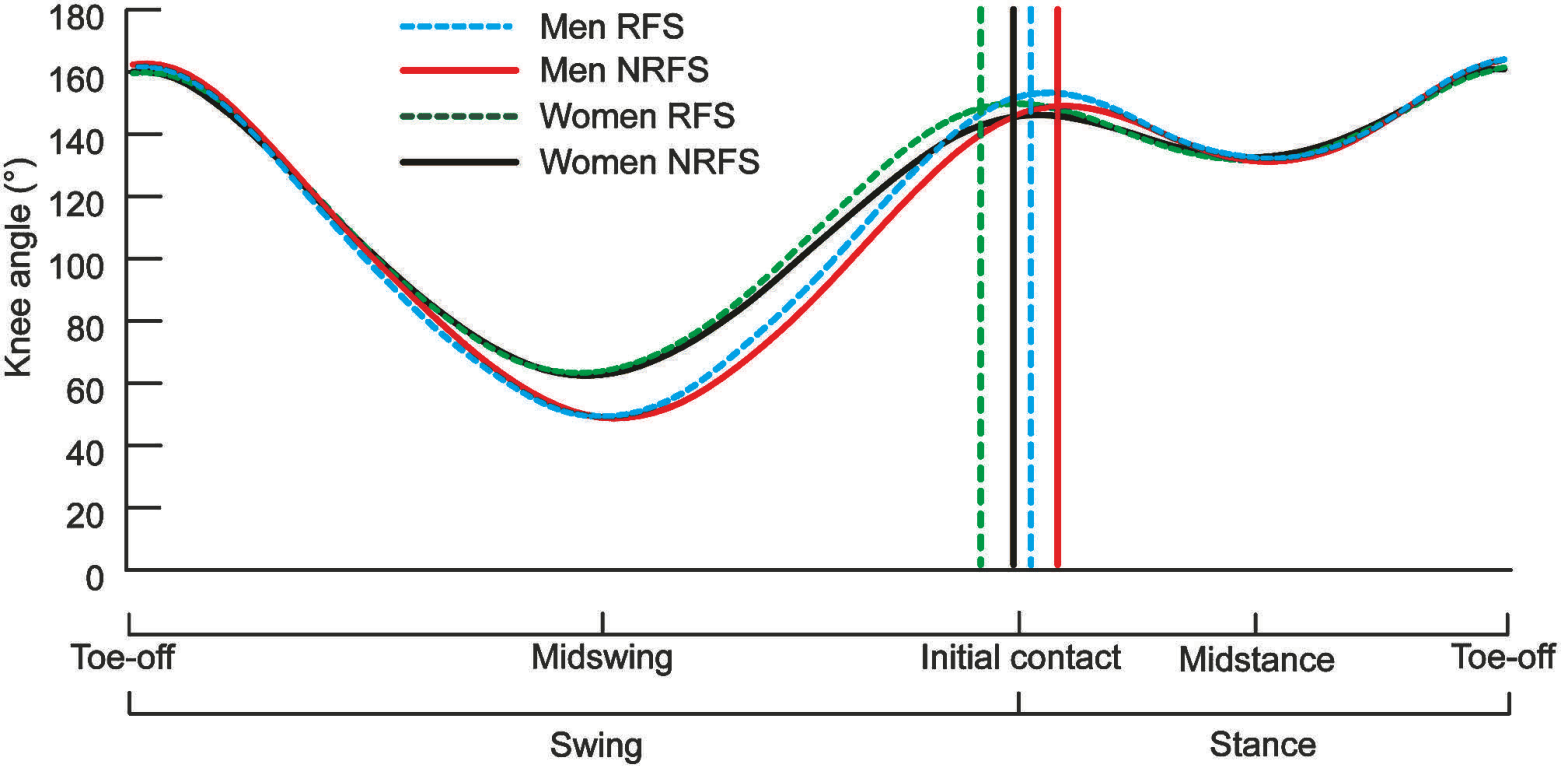
Knee angle during one complete gait cycle for RFS and NRFS in both men's and women's races during lap 3. Results are shown as means, with SD not indicated for any group for clarity (means and SDs are shown in **Tables 4**–**7** for knee angles at initial contact, midstance, toe-off and midswing, respectively). The vertical lines represent the mean initial contact times (as a percentage of the gait cycle) for each group indicated.

Descriptions of the variables analyzed in this study are presented in Table 1. All these variables were obtained using the 50 Hz cameras and used directly to calculate the values reported. When summed, the foot ahead, foot behind, flight distance and foot movement distances add up to step length; because it was not possible to measure participants' statures, which might have had an effect on spatial values, each of the four distances was also normalized as a proportion (%) of total step length for comparison purposes. Each athlete's knee angular data were interpolated to 101 points using a cubic spline to equalize the length of the gait cycle for presentation in Figure 1 (these interpolated data were not used to calculate the knee angle results reported). Joint angular data were averaged between left and right sides, rounded to the nearest integer, and have been presented in this study at specific events of the gait cycle, as defined below:

- Initial contact – the first visible instant during stance where the athlete's foot clearly contacted the ground.
- Midstance – the instant during stance where the athlete's foot center of mass was directly below the CM (i.e., in the horizontal anteroposterior direction).
- Midswing (knee angle only): the instant during swing where the athlete's knee was at its most flexed position (i.e., the minimum knee angle).
- Toe-off: the last visible instant during stance before the foot left the ground.

**Table 1:** Variables analyzed in the study and their description.

**Variable name**	**Description**
Running speed (km/h)	The mean horizontal speed during a complete gait cycle
Step length (m)	The distance between successive foot contacts from a specific event on the gait cycle on a particular foot (e.g., toe-off) to the equivalent event on the other foot
Cadence (Hz)	Calculated by dividing horizontal speed by step length (Mero and Komi, 1994)
Contact time (s)	The time duration from initial contact to toe-off
Flight time (s)	The time duration from toe-off of one foot to the initial contact of the opposite foot (Padulo et al., 2014)
Flight distance (m)	The distance the CM traveled during flight (from the instant of toe-off on a particular foot to the instant of initial contact on the other foot) (Hunter et al., 2004)
Foot ahead distance (m)	The distance from the center of mass of the landing foot to the CM
Foot behind distance (m)	The distance from the center of mass of the toe-off foot to the CM
Foot movement distance (m)	The distance the foot center of mass moved from its horizontal position at initial contact to toe-off
Overstriding distance (m)	The distance between the horizontal coordinate of the contact leg knee and the ipsilateral ankle, where larger distances indicated that the ankle landed farther in front of the knee
Hip angle (°)	The sagittal plane angle between the trunk and thigh segments (180° in the anatomical standing position)
Knee angle (°)	The sagittal plane angle between the thigh and lower leg segments (180° in the anatomical standing position)
Ankle angle (°)	The sagittal plane angle between the lower leg and foot segments, calculated in a clockwise direction (110° in the anatomical standing position) (Cairns et al., 1986)
Shoulder angle (°)	The sagittal plane angle between the trunk and upper arm (0° in the anatomical standing position; negative values for the shoulder therefore indicated a hyperextended position)
Elbow angle (°)	The sagittal plane angle between the upper arm and forearm (180° in the anatomical standing position)
Pelvic rotation (°)	The transverse plane angle calculated using the left and right hip joint coordinates
Shoulder girdle rotation (°)	The transverse plane angle calculated using the left and right shoulder joint coordinates

### Statistics

Statistical analyses were conducted using SPSS Statistics 26 (IBM SPSS, Inc., Chicago, IL). Normality of data was tested using the Shapiro-Wilk's test. Overall interactions between footstrike patterns, sex and lap were measured using a three-way mixed analysis of variance (ANOVA), with two-way analyses also found. Because each condition consisted of two groups, spatiotemporal, kinematic and performance variables were compared between men and women, and between RFS and NRFS using independent *t*-tests with adjustments made if Levene's test for equality of variances was less than 0.05, whereas within-athlete comparisons between laps were conducted using dependent *t*-tests (Field, 2009). An alpha level of 5% was set for all tests. To control for the number of statistical tests conducted, effect sizes were calculated using Cohen's *d* where differences were found within comparisons (Cohen, 1988) and considered to be either trivial (*d* ≤ 0.20), small (0.21 – 0.60), moderate (0.61–1.20), large (1.21–2.00), or very large (2.01–4.00) (Hopkins et al., 2009). Pearson's product moment correlation coefficient (*r*) was used to find associations separately within each sample of 28 men and 28 women, and considered to be small (*r* = 0.10–0.29), moderate (0.30–0.49), large (0.50–0.69) or very large (≥0.70) (Hopkins et al., 2009). Only those correlations that were large or very large were considered significant (in addition to an alpha value < 0.05).

## Results

The mean PR times (h:min:s) before competition for the 28 men and 28 women analyzed were 2:11:58 (± 4:42) and 2:31:44 (± 6:24), respectively. The mean finishing time for the men was 2:17:21 (± 5:09), whereas for the women, it was 2:37:29 (± 5:59); two men and three women ran PRs in this particular event. The men had faster PR and finishing times (*p* < 0.001, *d* ≥ 3.52). Both men and women had normally distributed finishing times. There was no difference in PR or finishing times between RFS and NRFS in either race, and there was no difference in running speed for the analyzed section between RFS and NRFS during either lap 3 or lap 4 in either race (Table 2). There were also no differences for step length or cadence between RFS and NRFS (Table 2), although large differences were found for overstriding distance on both laps, as well as differences for foot ahead distance on both laps and foot behind distance for women on lap 4 only (Table 3). Given that men are generally taller than women, it was not surprising that absolute distances were greater (Table 3), but in proportional terms men relied more on flight distance than women for overall step length (Table 4). Between laps 3 and 4, RFS and NRFS men experienced decreases in speed and step length that were large or moderate, whereas the decreases in these variables for women were smaller in general (Table 2); there was an interaction found between sex and lap for running speed (*p* = 0.035), in that men slowed more than women. Similarly, both RFS and NRFS men experienced large increases in contact time between laps, whereas only the NRFS women experienced a small increase (Table 2). In terms of components of step length, flight distances decreased between laps 3 and 4 for RFS and NRFS men (moderate effect size) and for NRFS women (small effect size); changes in foot ahead distance were found in RFS men only, although moderate decreases in foot behind distance were found in RFS and NRFS men, with small changes in NRFS women (Table 3).

**Table 2:** Mean ± SD values for key spatiotemporal variables.

	**Men**			**Women**		
	**RFS**	**NRFS**	**All**	**RFS**	**NRFS**	**All**
**Speed (km/h)**						
Lap 3	17.30 (± 1.32)	17.18 (± 0.87)	17.24 (± 1.10)	14.56 (± 1.03)	14.69 (± 0.95)	14.63^**^ (± 0.98)
Lap 4	15.66^b^ (± 1.54)	15.46^c^ (± 1.24)	15.56^c^ (± 1.38)	13.99^a^ (± 1.01)	13.84^b^ (± 1.48)	13.92^**^^b^ (± 1.24)
**Step length (m)**						
Lap 3	1.56 (± 0.10)	1.55 (± 0.10)	1.56 (± 0.10)	1.29 (± 0.10)	1.28 (± 0.11)	1.28^**^ (± 0.10)
Lap 4	1.45^b^ (± 0.11)	1.43^b^ (± 0.11)	1.44^b^ (± 0.11)	1.25^a^ (± 0.10)	1.22^a^ (± 0.15)	1.24^**^^a^ (± 0.13)
**Cadence (Hz)**						
Lap 3	3.07 (± 0.12)	3.08 (± 0.15)	3.07 (± 0.13)	3.15 (± 0.15)	3.19 (± 0.20)	3.17^*^ (± 0.17)
Lap 4	3.00^a^ (± 0.15)	3.00^a^ (± 0.16)	3.00^a^ (± 0.15)	3.10^a^ (± 0.13)	3.17 (± 0.21)	3.14^*^ (± 0.17)
**Contact time (s)**						
Lap 3	0.22 (± 0.01)	0.21^†^ (± 0.01)	0.22 (± 0.01)	0.24 (± 0.02)	0.22^†^ (± 0.01)	0.23^*^ (± 0.02)
Lap 4	0.24^c^ (± 0.02)	0.23^c^ (± 0.02)	0.23^c^ (± 0.02)	0.25 (± 0.02)	0.23^†*a*^ (± 0.02)	0.24^a^ (± 0.02)
**Flight time (s)**						
Lap 3	0.10 (± 0.01)	0.11^†^ (± 0.01)	0.11 (± 0.01)	0.08 (± 0.02)	0.09 (± 0.02)	0.09^**^ (± 0.02)
Lap 4	0.10 (± 0.02)	0.11 (± 0.02)	0.10 (± 0.02)	0.08 (± 0.02)	0.09 (± 0.02)	0.08^*^ (± 0.02)

*RFS, Rearfoot strikers; NRFS, Non-rearfoot strikers*.

† *Differences between RFS and NRFS were moderate (p < 0.05, d = 0.61–1.20)*.

* *Differences between men and women were moderate (p < 0.05, d = 0.61–1.20)*.

** *Differences between men and women were large or very large (p < 0.05, d > 1.21)*.

a *Differences between laps 3 and 4 were small (p < 0.05, d = 0.21–0.60)*.

b *Differences between laps 3 and 4 were moderate (p < 0.05, d = 0.61–1.20)*.

c *Differences between laps 3 and 4 were large or very large (p < 0.05, d > 1.21)*.

**Table 3:** Mean ± SD values for step length component variables and overstriding distance.

	**Men**			**Women**		
	**RFS**	**NRFS**	**All**	**RFS**	**NRFS**	**All**
**Flight distance (m)**						
Lap 3	0.60 (± 0.08)	0.64 (± 0.07)	0.62 (± 0.08)	0.42 (± 0.10)	0.47 (± 0.10)	0.44^**^ (± 0.10)
Lap 4	0.52^b^ (± 0.10)	0.56^b^ (± 0.10)	0.54^b^ (± 0.10)	0.39 (± 0.10)	0.43^a^ (± 0.11)	0.41^**^^a^ (± 0.11)
**Foot ahead (m)**						
Lap 3	0.37 (± 0.03)	0.33^‡^ (± 0.04)	0.35 (± 0.04)	0.32 (± 0.02)	0.30^‡^ (± 0.03)	0.31^**^ (± 0.03)
Lap 4	0.35^b^ (± 0.02)	0.32^‡^ (± 0.02)	0.33^a^ (± 0.03)	0.32 (± 0.03)	0.29^†^ (± 0.03)	0.31^*^ (± 0.03)
**Foot behind (m)**						
Lap 3	0.47 (± 0.03)	0.47 (± 0.04)	0.47 (± 0.04)	0.43 (± 0.04)	0.42 (± 0.03)	0.43^**^ (± 0.03)
Lap 4	0.45^b^ (± 0.03)	0.44^b^ (± 0.02)	0.45^b^ (± 0.02)	0.43 (± 0.03)	0.41^†*a*^ (± 0.02)	0.42^**^ (± 0.03)
**Foot movement (m)**						
Lap 3	0.11 (± 0.01)	0.11 (± 0.01)	0.11 (± 0.01)	0.12 (± 0.02)	0.10^†^ (± 0.01)	0.11 (± 0.02)
Lap 4	0.12^b^ (± 0.01)	0.11^†^ (± 0.01)	0.12 (± 0.01)	0.11 (± 0.02)	0.10^‡^ (± 0.01)	0.10^*^ (± 0.02)
**Overstriding distance (m)**						
Lap 3	0.05 (± 0.02)	0.02^‡^ (± 0.02)	0.03 (± 0.03)	0.04 (± 0.02)	0.00^‡^ (± 0.02)	0.02 (± 0.02)
Lap 4	0.04 (± 0.02)	0.01^‡^ (± 0.01)	0.03 (± 0.02)	0.03 (± 0.02)	0.00^‡^ (± 0.02)	0.02 (± 0.02)

*RFS, Rearfoot strikers; NRFS, Non-rearfoot strikers*.

† *Differences between RFS and NRFS were moderate (p < 0.05, d = 0.61–1.20)*.

‡ *Differences between RFS and NRFS were large (p < 0.05, d = 0.61–1.20)*.

* *Differences between men and women were moderate (p < 0.05, d = 0.61–1.20)*.

** *Differences between men and women were large or very large (p < 0.05, d > 1.21)*.

a *Differences between laps 3 and 4 were small (p < 0.05, d = 0.21–0.60)*.

b *Differences between laps 3 and 4 were moderate (p < 0.05, d = 0.61–1.20)*.

**Table 4:** Mean ± SD values for step length components expressed as a percentage of total step length.

	**Men**			**Women**		
	**RFS**	**NRFS**	**All**	**RFS**	**NRFS**	**All**
**Flight distance (% of total step length)**						
Lap 3	38.4 (± 3.1)	41.3^†^ (± 3.5)	39.8 (± 3.6)	32.1 (± 5.8)	36.5^†^ (± 5.0)	34.3^*^ (± 5.8)
Lap 4	35.8^b^ (± 4.7)	39.0 (± 4.3)	37.4^a^ (± 4.7)	30.8 (± 5.7)	34.6 (± 5.4)	32.7^*^^a^ (± 5.8)
**Foot ahead (% of total step length)**						
Lap 3	23.9 (± 1.6)	21.4^‡^ (± 1.6)	22.6 (± 2.0)	25.3 (± 1.5)	23.1^†^ (± 2.5)	24.2^*^ (± 2.3)
Lap 4	24.3 (± 1.8)	22.3^†^ (± 1.6)	23.3 (± 1.9)	25.9 (± 2.3)	23.9^†^ (± 2.4)	24.9^*^ (± 2.5)
**Foot behind (% of total step length)**						
Lap 3	30.3 (± 1.4)	30.4 (± 2.2)	30.4 (± 1.8)	33.3 (± 3.3)	32.5 (± 2.4)	32.9^*^ (± 2.8)
Lap 4	31.4 (± 2.7)	31.1 (± 2.3)	31.2 (± 2.5)	34.1 (± 2.7)	33.6 (± 3.1)	33.9^*^ (± 2.8)
**Foot movement (% of total step length)**						
Lap 3	7.3 (± 1.0)	6.9 (± 1.0)	7.1 (± 1.0)	9.2 (± 1.8)	7.8^†^ (± 1.2)	8.5^*^ (± 1.7)
Lap 4	8.6^b^ (± 1.2)	7.6^†^^b^ (± 0.9)	8.1^b^ (± 1.1)	9.2 (± 1.6)	7.9^†^ (± 1.1)	8.5 (± 1.5)

*RFS, Rearfoot strikers; NRFS, Non-rearfoot strikers*.

† *Differences between RFS and NRFS were moderate (p < 0.05, d = 0.61–1.20)*.

‡ *Differences between RFS and NRFS were large (p < 0.05, d = 0.61–1.20)*.

* *Differences between men and women were moderate (p < 0.05, d = 0.61–1.20)*.

a *Differences between laps 3 and 4 were small (p < 0.05, d = 0.21–0.60)*.

b *Differences between laps 3 and 4 were moderate (p < 0.05, d = 0.61–1.20)*.

At initial contact, RFS had more extended knees, dorsiflexed ankles and hyperextended shoulders than NRFS in the men's race, whereas in the women's race, RFS had more flexed hips and extended knees than NRFS (Table 5); each group's mean knee angle throughout the gait cycle are shown in Figure 1. Fewer differences were found between RFS and NRFS in both sexes at midstance and toe-off (Tables 6, 7, respectively), although RFS had more flexed shoulders at toe-off than NRFS in the women's race. There were interactions found between sex and footstrike pattern for hip angle at initial contact, and hip, ankle and shoulder angles at midstance (*p* ≤ 0.046). Men had greater pelvic rotation than women, whereas women had greater shoulder girdle rotation (Table 8). During midswing, there were no differences in knee flexion angle between RFS and NRFS during either lap in men or women (Table 8), but men had lower knee angles during this phase. Indeed, knee flexion angle during midswing was the only joint angle to change between laps 3 and 4, increasing in both RFS and NRFS men (no change for women) (Table 8). The correlations between the most important spatiotemporal variables, as well as knee flexion because of its change in men from lap 3 to 4, were included in Table 9. Knee flexion was strongly correlated with step length and flight distance in both sexes (Table 9); no other joint angles consistently correlated with key spatiotemporal variables across laps or sexes, and none found were large. There were very large correlations between speed and step length, but not cadence. Similarly, flight distance was strongly correlated with speed and step length, but not with cadence (Table 9). Greater overstriding distances were associated with larger foot ahead distances in both men and women (Table 9), but not with speed, step length or cadence.

**Table 5:** Mean ± SD values for joint angles at initial contact.

	**Men**			**Women**		
	**RFS**	**NRFS**	**All**	**RFS**	**NRFS**	**All**
**Hip (°)**						
Lap 3	143 (± 5)	142 (± 4)	142 (± 4)	143 (± 5)	147^†^ (± 4)	145 (± 5)
Lap 4	144 (± 3)	144 (± 6)	144 (± 5)	143 (± 4)	148^†^ (± 5)	146 (± 5)
**Knee (°)**						
Lap 3	152 (± 4)	147^‡^ (± 3)	149 (± 4)	150 (± 5)	147^†^ (± 3)	148 (± 4)
Lap 4	151 (± 4)	147^†^ (± 3)	149 (± 4)	150 (± 4)	147 (± 3)	148 (± 4)
**Ankle (°)**						
Lap 3	100 (± 4)	104^‡^ (± 2)	102 (± 4)	97 (± 3)	99 (± 3)	98^*^ (± 3)
Lap 4	101 (± 5)	105^†^ (± 4)	103 (± 5)	98 (± 3)	99 (± 4)	99^*^ (± 4)
**Shoulder (°)**						
Lap 3	−50 (± 6)	−43^‡^ (± 5)	−47 (± 6)	−52 (± 7)	−48 (± 7)	−50 (± 7)
Lap 4	−51 (± 5)	−46^†^ (± 6)	−48 (± 6)	−52 (± 6)	−49 (± 8)	−51 (± 7)
**Elbow (°)**						
Lap 3	70 (± 11)	70 (± 12)	70 (± 11)	65 (± 11)	67 (± 20)	66 (± 16)
Lap 4	68 (± 10)	67 (± 11)	68 (± 10)	65 (± 10)	67 (± 16)	66 (± 13)

*There were no significant effects found for laps*.

*RFS, Rearfoot strikers; NRFS, Non-rearfoot strikers*.

† *Differences between RFS and NRFS were moderate (p < 0.05, d = 0.61–1.20)*.

‡ *Differences between RFS and NRFS were large (p < 0.05, d = 0.61–1.20)*.

* *Differences between men and women were moderate (p < 0.05, d = 0.61–1.20)*.

**Table 6:** Mean ± SD values for joint angles at midstance.

	**Men**			**Women**		
	**RFS**	**NRFS**	**All**	**RFS**	**NRFS**	**All**
**Hip (°)**						
Lap 3	151 (± 4)	151 (± 4)	151 (± 4)	150 (± 6)	154 (± 4)	152 (± 5)
Lap 4	153 (± 4)	153 (± 5)	153 (± 4)	150 (± 5)	155^†^ (± 4)	152 (± 5)
**Knee (°)**						
Lap 3	131 (± 4)	131 (± 4)	131 (± 4)	131 (± 4)	131 (± 3)	131 (± 4)
Lap 4	132 (± 3)	131 (± 4)	132 (± 4)	131 (± 4)	132 (± 3)	131 (± 4)
**Ankle (°)**						
Lap 3	81 (± 3)	83 (± 3)	82 (± 3)	81 (± 2)	81 (± 2)	81 (± 2)
Lap 4	81 (± 2)	84^†^ (± 2)	83 (± 2)	81 (± 2)	81 (± 1)	81^*^ (± 2)
**Shoulder (°)**						
Lap 3	−28 (± 5)	−24^†^ (± 5)	−26 (± 5)	−28 (± 4)	−28 (± 7)	−28 (± 6)
Lap 4	−28 (± 5)	−25 (± 4)	−26 (± 5)	−27 (± 5)	−28 (± 5)	−28 (± 5)
**Elbow (°)**						
Lap 3	73 (± 8)	70 (± 13)	71 (± 10)	71 (± 11)	73 (± 17)	72 (± 14)
Lap 4	72 (± 9)	70 (± 11)	71 (± 10)	70 (± 11)	74 (± 14)	72 (± 12)

*There were no significant effects found for laps*.

*RFS, Rearfoot strikers; NRFS, Non-rearfoot strikers*.

† *Differences between RFS and NRFS were moderate (p < 0.05, d = 0.61–1.20)*.

* *Differences between men and women were moderate (p < 0.05, d = 0.61–1.20)*.

**Table 7:** Mean ± SD values for joint angles at toe-off.

	**Men**			**Women**		
	**RFS**	**NRFS**	**All**	**RFS**	**NRFS**	**All**
**Hip (°)**						
Lap 3	192 (± 4)	192 (± 4)	192 (± 4)	190 (± 4)	193 (± 4)	191 (± 4)
Lap 4	191 (± 3)	191 (± 4)	191 (± 3)	190 (± 4)	192 (± 3)	191 (± 4)
**Knee (°)**						
Lap 3	162 (± 4)	163 (± 4)	163 (± 4)	160 (± 4)	160 (± 3)	160^*^ (± 4)
Lap 4	163 (± 3)	162 (± 5)	163 (± 4)	159 (± 4)	161 (± 5)	160^*^ (± 4)
**Ankle (°)**						
Lap 3	126 (± 6)	128 (± 6)	127 (± 6)	123 (± 6)	124 (± 4)	124^*^ (± 5)
Lap 4	126 (± 6)	128 (± 6)	127 (± 6)	123 (± 5)	125 (± 4)	124^*^ (± 5)
**Shoulder (°)**						
Lap 3	28 (± 5)	25 (± 6)	27 (± 5)	29 (± 4)	24^†^ (± 6)	27 (± 6)
Lap 4	27 (± 4)	24 (± 4)	26 (± 5)	30 (± 5)	25^†^ (± 5)	27 (± 6)
**Elbow (°)**						
Lap 3	57 (± 9)	54 (± 10)	55 (± 10)	58 (± 9)	58 (± 16)	58 (± 13)
Lap 4	57 (± 9)	55 (± 10)	56 (± 9)	56 (± 8)	58 (± 12)	57 (± 10)

*There were no significant effects found for laps*.

*RFS, Rearfoot strikers; NRFS, Non-rearfoot strikers*.

* *Differences between men and women were moderate (p < 0.05, d = 0.61–1.20)*.

† *Differences between RFS and NRFS were moderate (p < 0.05, d = 0.61–1.20)*.

**Table 8:** Mean ± SD values for maximum pelvic and shoulder girdle rotation and minimum knee angle (flexion) during midswing.

	**Men**			**Women**		
	**RFS**	**NRFS**	**All**	**RFS**	**NRFS**	**All**
**Pelvic rotation (°)**						
Lap 3	11 (± 3)	11 (± 4)	11 (± 4)	6 (± 3)	5 (± 2)	5^**^ (± 2)
Lap 4	10 (± 3)	10 (± 5)	10 (± 4)	5 (± 2)	4 (± 2)	5^**^ (± 2)
**Shoulder girdle rotation (°)**						
Lap 3	15 (± 3)	17 (± 3)	16 (± 3)	19 (± 3)	19 (± 3)	19^*^ (± 3)
Lap 4	16 (± 3)	17 (± 3)	16 (± 3)	19 (± 4)	19 (± 3)	19^*^ (± 3)
**Knee angle during midswing (°)**						
Lap 3	50 (± 6)	50 (± 6)	50 (± 6)	63 (± 10)	63 (± 11)	63^*^ (± 10)
Lap 4	54^b^ (± 6)	55^b^ (± 7)	55^b^ (± 7)	64 (± 11)	65 (± 12)	65^*^ (± 11)

*There were no significant effects found for footstrike pattern*.

*RFS, Rearfoot strikers; NRFS, Non-rearfoot strikers*.

* *Differences between men and women were moderate (p < 0.05, d = 0.61–1.20)*.

** *Differences between men and women were large or very large (p < 0.05, d > 1.21)*.

b *Differences between laps 3 and 4 were moderate (p < 0.05, d = 0.61–1.20)*.

**Table 9:** Correlation analysis of key variables in World Championship marathon runners during Laps 3 and 4.

		**Step length**	**Cadence**	**Foot ahead**	**Foot behind**	**Flight distance**
**Men**						
Speed	Lap 3	***r* = 0.76**	*r* = 0.35	*r* = 0.41	***r* = 0.50**	***r* = 0.55**
	Lap 4	***r* = 0.81**	***r* = 0.52**	*r* = 0.22	*r* = 0.28	***r* = 0.74**
Step length	Lap 3		*r* = −0.34	***r* = 0.57**	***r* = 0.65**	***r* = 0.71**
	Lap 4		*r* = −0.07	*r* = 0.36	*r* = 0.30	***r* = 0.90**
Cadence	Lap 3			*r* = −0.25	*r* = −0.19	*r* = −0.23
	Lap 4			*r* = −0.19	*r* = 0.02	*r* = −0.04
Knee flexion	Lap 3	***r* = −0.66**	*r* = 0.42	*r* = −0.38	*r* = −0.19	***r* = −0.59**
	Lap 4	***r* = −0.79**	***r* = 0.50**	*r* = −0.27	*r* = −0.19	***r* = −0.73**
Overstriding distance	Lap 3	*r* = 0.44	*r* = −0.07	***r* = 0.81**	*r* = 0.15	*r* = 0.05
	Lap 4	*r* = 0.39	*r* = −0.15	***r* = 0.83**	*r* = 0.09	*r* = 0.12
**Women**						
Speed	Lap 3	***r* = 0.73**	*r* = 0.14	*r* = 0.29	*r* = 0.07	***r* = 0.68**
	Lap 4	***r* = 0.83**	*r* = 0.07	*r* = 0.44	*r* = 0.30	***r* = 0.86**
Step length	Lap 3		***r* = −0.57**	*r* = 0.38	*r* = 0.37	***r* = 0.84**
	Lap 4		*r* = −0.49	***r* = 0.53**	***r* = 0.54**	***r* = 0.86**
Cadence	Lap 3			*r* = −0.21	*r* = −0.45	*r* = −0.40
	Lap 4			*r* = −0.29	***r* = −0.50**	*r* = −0.38
Knee flexion	Lap 3	***r* = −0.83**	*r* = 0.49	*r* **=** −0.09	*r* = 0.02	***r* = −0.88**
	Lap 4	***r* = −0.86**	***r* = 0.52**	*r* = −0.34	*r* = −0.27	***r* = −0.86**
Overstriding	Lap 3	*r* = 0.11	*r* = −0.18	***r* = 0.65**	*r* = 0.23	*r* = −0.22
distance	Lap 4	*r* = 0.43	*r* = −0.33	***r* = 0.70**	***r* = 0.51**	*r* = 0.11

*Correlations were significant at p < 0.05 and r ≥ 0.50 (shown in bold)*.

## Discussion

The aim of this study was to analyze spatiotemporal and joint kinematic variables in male and female marathon RFS and NRFS runners across the last two 10.5-km laps at the 2017 IAAF World Championships. The first hypothesis, that RFS would have longer steps and lower cadences than NRFS, was rejected as there were no differences in step length or cadence at the same running speed. Regarding the related hypothesized joint angular differences at initial contact, both NRFS men and women had less knee extension, but whereas the NRFS men had more plantarflexed ankles at initial contact, NRFS the women did not; however, the NRFS women did have less flexed hips. Although the effect of these joint angular differences did not manifest as differences in the two key spatiotemporal variables of step length and cadence, they did result in a greater foot ahead proportion for RFS men and women on both laps, and greater flight distance proportions for NRFS men and women on lap 3. Therefore, given that step length is the same, the main differences in running technique between RFS and NRFS world-class marathon runners are a greater reliance on foot ahead distance in RFS, achieved in both sexes with more extended knees, and that NRFS athletes rely more on flight distance. In theory, a greater foot ahead distance results in more braking forces (Moore, 2016), and although these could not be measured in this study, there were nonetheless no performance differences within these groups. Indeed, it should be noted that NRFS athletes still landed with their foot just over 0.30 m ahead of the CM, and it is possible that the absolute foot ahead differences between RFS and NRFS of 0.02–0.04 m were too small to be meaningful in that regard. Similarly, although RFS athletes of both sexes had greater mean overstriding distances at initial contact, these were only 0.03–0.04 m greater than in NRFS and, given there were no correlations between overstriding distance and the key performance variables of speed, step length and cadence, such differences might have been insufficient for any competitive advantage. Coaches should therefore note that encouraging marathon runners to convert from RFS to NRFS is likely to result in few if any benefits to performance, especially as continuous NRFS can lead to considerable fatigue in the lower limb's contractile properties (Peltonen et al., 2012; Baggaley et al., 2017) and explains why many NRFS to switch to RFS in the later stages of the race (Hanley et al., 2019).

As stated above, running speed is the product of step length and cadence although, within this sample of elite-standard athletes, step length was much more strongly correlated with speed. This does not mean that an appropriately high cadence (>3 Hz) is not important in achieving competitive running speeds, but rather signifies that cadence varied little amongst this relatively homogenous group and thus was not a distinguishing factor for performance. Instead, the importance of step length shows that it is the chief differentiator of marathon running ability, and was strongly correlated with flight distance. Indeed, although step length and flight distance decreased from lap 3 to 4, these variables' association with speed increased on lap 4. A trade-off between longer steps and reduced cadences is normal in running (Heiderscheit et al., 2011), although the negative correlations between foot ahead distance and cadence were small and not indicative of meaningful overstriding in this cohort of well-trained athletes. The movement of the recovery leg during swing was important as those athletes who flexed their knees more had longer steps and flight distances and, in women, faster running speeds. These associations were very large during lap 4 and highlighted the role of the flexed knee during midswing in reducing the energy requirements of the recovery leg (Elliot and Ackland, 1981) that aids with an improved flight phase (Smith and Hanley, 2013). However, it is possible that increased knee flexion is an outcome of faster running because of the rapid forward movement of the thigh during swing (Mann and Hagy, 1980), rather than a cause of it. Furthermore, it is possible that the increased correlation values for knee flexion on lap 4, as well as those for step length and flight distance, occurred to some extent because of greater ranges in the data during the last lap, itself resulting from a greater separation of athletes because of fatigue.

It was unsurprising that men had greater absolute values for running speed and for those variables influenced by stature, such as step length and its components (apart from foot movement on lap 3). Women had greater cadences than men on both laps, mostly because of shorter flight times, which in turn meant that women relied less on flight distance for total step length. Given its importance to running speed, it was noticeable that the largest absolute sex-based difference for any component of step length was for flight distance (longer by 0.18 and 0.13 m in men on laps 3 and 4, respectively). Women compensated for the smaller contribution of flight distance with longer proportions of foot ahead and foot behind distances on both laps. These small proportional differences were manifested in very few joint angular differences overall, although men had more plantarflexed ankles at initial contact (both laps), midstance (lap 4 only) and toe-off (both laps), and more extended knees at toe-off on both laps. Interestingly, given its strong association with flight distance and step length, knee flexion during midswing was greater in men than women and thus is one of the few technical sex-based differences, although as noted above, this might be an outcome of men's faster running speeds rather than a contributor.

The second hypothesis was that those differences found between RFS and NRFS would be similar for men and women. As mentioned above, there were no differences in step length or cadence in RFS and NRFS for either men or women, although the differences at initial contact in ankle angle between RFS and NRFS amongst men were not found in women, who had different hip flexion magnitudes instead (highlighted by the interaction between sex and footstrike pattern). This sex-based difference in ankle and hip joint angles between footstrike patterns was also found at midstance on lap 4. Additionally, in the men's race, RFS had more hyperextended shoulders at initial contact, whereas in the women's race, RFS had more flexed shoulders at toe-off, indicating that slightly different upper body movements are used by men and women to counterbalance the lower limbs' movements. This point was further demonstrated by how men had greater pelvic rotation and less shoulder girdle rotation than women. Overall, however, any differences (or absence of differences, which were more common) between RFS and NRFS were found in both men and women. These include no differences between midswing knee flexion values between RFS and NRFS. From a technical point of view, this demonstrates that RFS and NRFS techniques are mostly indistinguishable except at initial contact, and that differences between the sexes are greater than differences between footstrike patterns. Ultimately, RFS and NRFS have running techniques that are so similar (Ardigò et al., 1995; Gruber et al., 2013), with any differences so small that they are possibly meaningless with regard to effects on performance, that there seems little rationale to encourage marathon runners to run with any footstrike pattern other than what they already do habitually.

The third hypothesis, that running speed and associated spatiotemporal variables would decrease between the second-last and last laps because of fatigue, and that any differences between RFS and NRFS would be consistent across laps, was mostly supported. Speed decreases occurred predominantly in line with reduced step lengths, although the effect sizes were larger in men than women, which might be related to men's greater slowing down between laps 3 and 4. RFS and NRFS athletes in the men's race, and RFS women, had small reductions in cadence also, which resulted from longer contact times. One reason for reduced running speeds is a decline in effectiveness of the stretch-shortening cycle in the muscle-tendon unit (Komi, 2000), which is reflected in a reduction in the storage of elastic energy, leading to fatigue and an increased need for muscular work to maintain a given speed (Nicol et al., 1991). Rather than being able to increase muscular work when fatigued, athletes simply slow and this is largely because they cannot achieve the same step lengths as when unfatigued. A reduction in elastic energy storage is caused partially by an increase in transition time from stretch to shortening (i.e., between braking and push-off phases) (Nicol et al., 1991) and might have occurred in this sample as shown by their longer contact times, although such neuromuscular factors could not be measured in competition. More so than footstrike pattern, distance run (and presumably the fatigue that accrues because of it) was unsurprisingly the main determinant of differences in spatiotemporal variables between laps. Maintaining step length and cadence as close as possible to the magnitudes achieved when running at faster speeds (as on lap 3) avoids decreases in running speed, with the maintenance of a long step length the more decisive of the two. Notwithstanding the need for highly developed cardiovascular and energy systems, being able to achieve this results to some extent from training the fatigue resistance of muscle-tendon units, particularly in the lower limb. The finding in previous research that many NRFS marathon runners switch to RFS by the end of the marathon (Larson et al., 2011; Hanley et al., 2019) suggests that many do not have fatigue resistance in the ankle plantarflexors necessary to retain a more anterior striking footstrike pattern (Peltonen et al., 2012; Baggaley et al., 2017).

The largest contributor to shortened step lengths was reduced flight distances (by a mean of 0.08 and 0.03 m in men and women, respectively), whereas no other contributor to step length decreased by more than 0.02 m. Flight distance proportion was one of the few spatiotemporal variables that was not consistently different between RFS and NRFS on both laps; however, most variables did not change between laps (in that they did not differ between RFS and NRFS) and highlights how athletes who have developed either footstrike pattern maintained a consistent technique, despite decreases in speed, step length and cadence. Indeed, there were no changes in stance phase joint angles between laps 3 and 4. However, it was noteworthy that men's minimum knee flexion angles increased during midswing by 5°, a change that has previously been found in a fatiguing 10,000 m race (Elliot and Ackland, 1981), and which might have been a function of reduced running speed (Mann and Hagy, 1980), especially as it did not decrease in women who suffered smaller decreases in speed. Overall, adopting one specific footstrike pattern or the other did not protect against a deterioration of running speed or lead to a change in technique with distance run. Given the few differences between RFS and NRFS on both of the last two laps, that any changes that occurred were similar between both, and that these similarities were quite consistent between men and women, coaches and athletes are advised that there is no strong rationale to change footstrike pattern from what is naturally preferred in either sex.

The main strength of this study was that it was conducted in the highly ecologically valid setting of a major championship race, where the athletes ran using their habitual running style and no intervention was involved. This means that the results found are an accurate reflection of world-class marathon running techniques in the sample studied. However, because there were more RFS runners in both races (Hanley et al., 2019), the 28 athletes who formed the RFS sample were less representative of all RFS competitors than the 28 NRFS athletes were of theirs. Nonetheless, both RFS and NRFS samples within each race were well distributed, as shown by the absence of differences between PR or finishing times. The duration of the competition and the number of athletes competing meant that a sampling rate of 50 Hz was the most suitable for data collection, although the time between frames of 0.02 s means that caution must be taken in particular when considering differences in the temporal values between footstrike patterns, sexes and laps (all values presented were obtained using the original 50 Hz data, rather than from the interpolated data used to create Figure 1). Footstrike patterns was treated as a discrete variable, rather than as a continuous one that might be measured using footstrike angle, for example, and this might have prevented more footstrike effects being identified. For this study, the athletes were recorded on all four laps they completed, but only during the last two were athletes spread out enough to enable high-quality analysis; future research could try to analyze athletes at more distances during the marathon to further evaluate the changes that occur with fatigue, and to obtain anthropometric data that could allow for the calculation of spatial variables relative to stature.

## Conclusions

This was the first study to analyze world-class marathon runners of both sexes in competition in comparing the spatiotemporal and joint kinematic differences between RFS and NRFS. The most important finding from all analyses and comparisons was that RFS and NRFS have very similar running techniques, with no differences in step length or cadence at the same running speed. Most joint angles in the upper and lower limbs were the same at key gait events, with most differences occurring at initial contact. This was unsurprising given that what differentiates RFS and NRFS is how the athletes land at initial contact, but even still, the absolute differences in overstriding, flight and foot ahead distances, and ankle, knee and hip joint angles were typically no more than 0.04 m and 5°, respectively. Although this is not meant to imply that RFS and NRFS techniques are identical, there is nonetheless little evidence to support coaching practices that aim to convert an athlete from one footstrike pattern to another. RFS and NRFS athletes of both sexes had similar reductions in speed, step length and cadence between laps 3 and 4, but there were few joint angular changes, showing that individual techniques were not considerably affected by fatigue. In terms of practical applications, coaches should note that the maintenance of a long step length, largely through maintaining a long flight distance, likely arises from training the fatigue resistance of muscle-tendon units, such as the ankle plantarflexors, alongside the development of a marathon runner's cardiovascular and energy systems capabilities.

## Data Availability Statement

The datasets presented in this article are not readily available because there is a risk of identifying individual athletes. Requests to access the datasets should be directed to Brian Hanley, b.hanley@leedsbeckett.ac.uk.

## Ethics Statement

The studies involving human participants were reviewed and approved by the Leeds Beckett University Carnegie School of Sport Research Ethics Advisory Group. The patients/participants provided their written informed consent to participate in this study.

## Author Contributions

BH, AB, and SM conceptualized and designed the study and wrote the manuscript. AB and SM arranged data collection during the World Championships marathon events as Project Director and Project Leader, respectively. BH collected and analyzed the video data. All authors read and approved the final manuscript.

## Conflict of Interest

The authors declare that the research was conducted in the absence of any commercial or financial relationships that could be construed as a potential conflict of interest.
